# Compound Volvulus: A Rare Case of Intestinal Obstruction

**DOI:** 10.7759/cureus.54936

**Published:** 2024-02-26

**Authors:** Ajit Sheoran, Sameeksha Shah, Shivani B Paruthy, Sushila Choudhary

**Affiliations:** 1 General Surgery, Vardhman Mahavir Medical College and Safdarjung Hospital, New Delhi, IND; 2 Surgery, Vardhman Mahavir Medical College and Safdarjung Hospital, New Delhi, IND

**Keywords:** emergency laparotomy, double close loop obstruction, compound volvulus, intestinal obstruction, ileosigmoid knotting

## Abstract

Compound volvulus or ileosigmoid knotting is an uncommon surgical emergency that causes intestinal obstruction. The sigmoid and ileum are mostly involved in this closed-loop intestinal obstruction. It is regarded as a rather uncommon cause of intestinal obstruction. It's important to distinguish between an ileosigmoid knot and a simple sigmoid volvulus since the management of the two is different.

CT and MRI are more helpful in the diagnosis than abdominal X-ray findings, which are not pathognomonic. After resuscitation, a patient with ileosigmoid knotting typically needs an emergency laparotomy. Different resectional and non-resectional surgical procedures may be employed depending on the viability of the ileum and sigmoid colon.

## Introduction

Ileosigmoid knotting (ISK) is a less-known cause of intestinal obstruction that results in a compound loop bowel obstruction by wrapping either the ileum or the sigmoid colon around the base of the other [1-3]. Although the prevalence is unknown, it is frequently observed in regions of the world where sigmoid volvulus (SV) is prevalent. In Eastern and Western nations, it makes up for 18-50% and 5-8% of SV cases, respectively [2-3]. With a high occurrence in the third to fifth decade, it is predominantly a male disease [3].

In 1845, Parker published the first ISK case [4]. The second case was reported by Kallio in 1932. In 1940, Paul wrote about the first instance of ISK from the Asian subcontinent. A very few cases have been described in the literature so far. Only 0-28% of cases could be detected preoperatively, making this surgical disease a diagnostic dilemma. Large gas-filled small and large bowel loops can be seen in the right mid and lower abdomen on an X-ray of the abdomen. Dilated, fluid-filled intestinal loops and free fluid in the pelvis are shown on ultrasound. The best diagnostic technique for making the correct diagnosis is computed tomography (CT) [1-4].

## Case presentation

A 45-year-old gentleman presented in our surgical emergency with generalized abdominal pain for six days and non-passage of stool and flatus for five days. The patient had no history of trauma or past treatment/surgical history.

**Figure 1:**
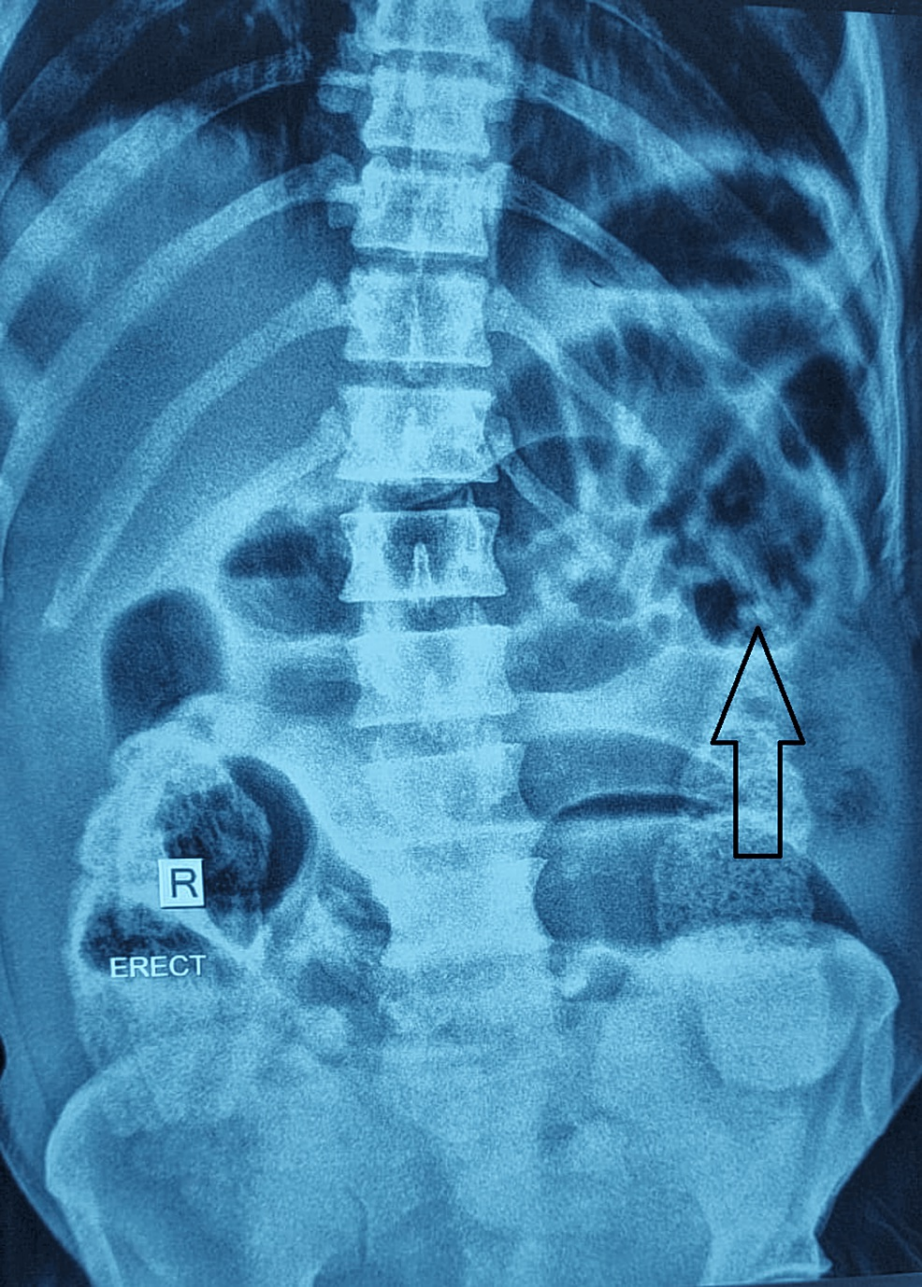
X-ray abdomen-dilated small and large bowel loops. Arrow showing dilated bowel loops.

On examination, the patient was conscious with BP 81/55 mmHg and pulse 114/min. The abdomen was distended with generalized tenderness, guarding, and absent bowel sounds. A nasogastric tube was inserted for proximal decompression and urethral catheterisation was done. Per-rectal examination shows a dilated rectum with no fecal staining. An erect X-ray of the abdomen (Figure 1) revealed dilated small and larger bowel loops. Ultrasonography (USG) abdomen revealed moderate ascites with internal septations and dilated bowel loops. Contrast-enhanced computed tomography (CECT) abdomen done from outside the hospital (blurred images) shows moderate ascites with closed loop obstruction, distal ileum twisting (360) around the sigmoid colon, and its mesentery suggestive of compound volvulus. CECT report shows of multiple sub-centimetric mesenteric nodes with fat stranding suggestive of mesenteric lymphadenopathy.

The patient was resuscitated with IV fluid therapy, broad-spectrum antibiotics, analgesics, and antiemetics. The patient was admitted to the intensive care unit for further monitoring and stabilization. Later he was started on ionotropic support and planned for emergency exploratory laparotomy. The pre-operative patient was conscious and oriented (BP 92/66 mmHg, PR 111/min on inotropic support, SpO2 97% on oxygen support at 4 L/min).

**Figure 2:**
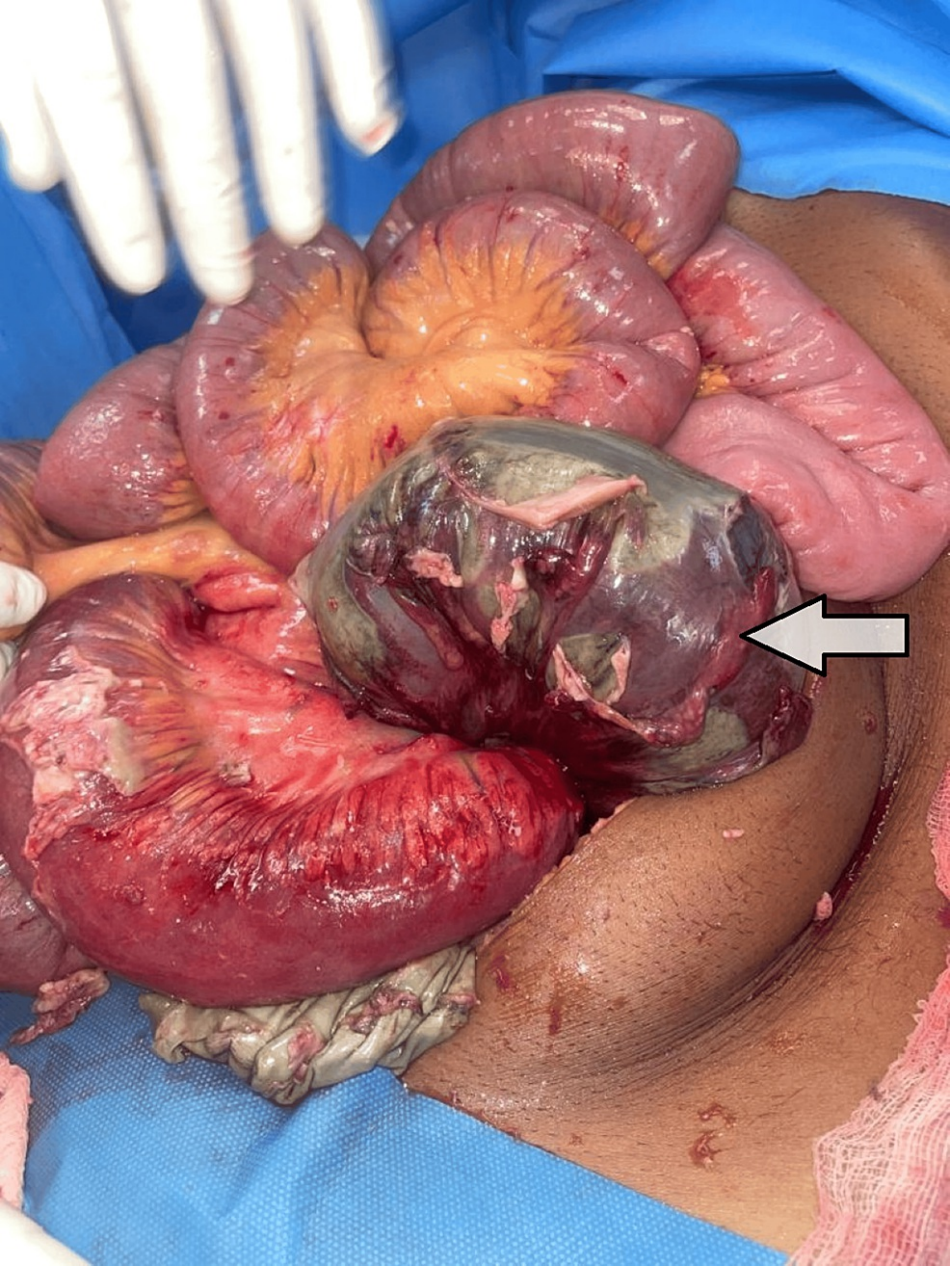
Laparotomy showing gangrenous sigmoid colon. Arrow showing gangrenous segment.

**Figure 3:**
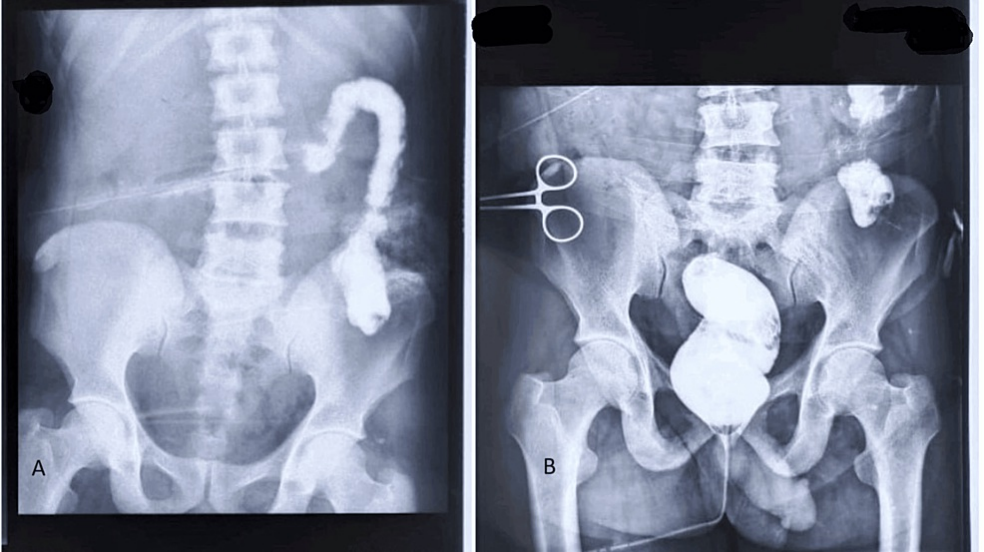
Preoperative distal loopogram with contrast inserted through transverse loop colostomy (Image A) and rectal contrast (Image B) Image A - colostomy, no dye leak. Image B - rectum with no dye leak.

**Figure 4:**
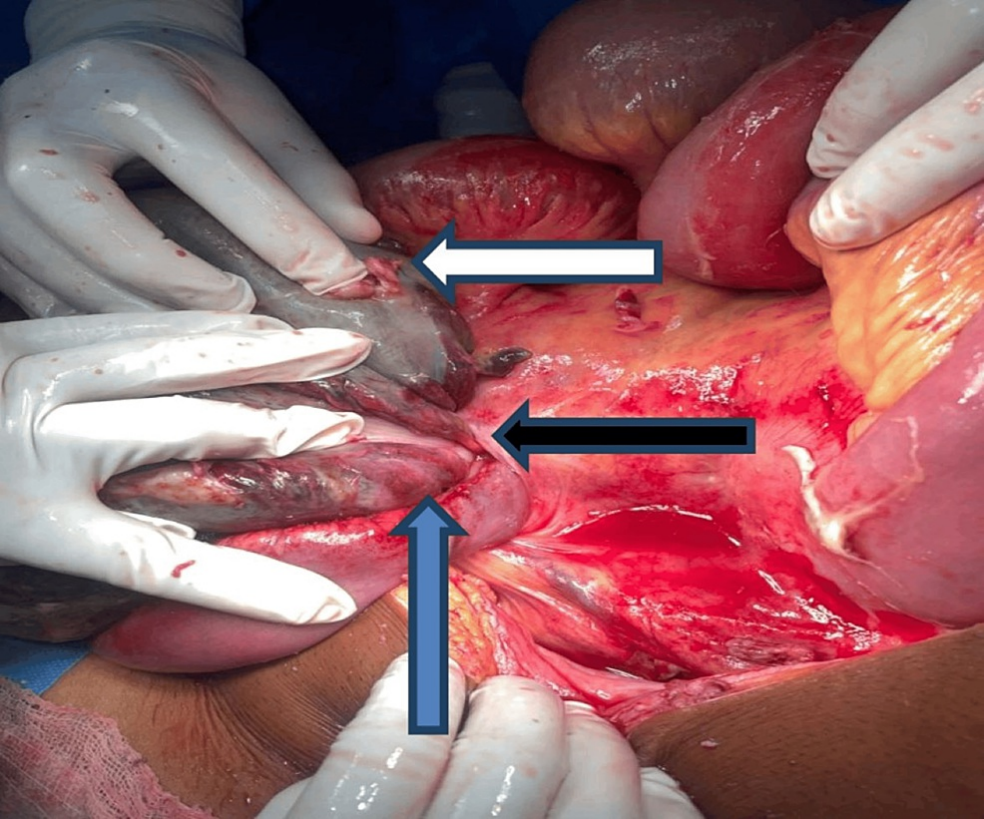
Laparotomy showing twisting of ileum and sigmoid colon with gangrenous changes. Blue arrow - sigmoid loop. White arrow - ileal loops. Black arrow - Axis of knotting.

Intraoperatively sigmoid colon was found gangrenous (Figure 2) and rotated 360 degrees anticlockwise along its axis. Distal ileum approximately one foot from ileocaecal junction (ICJ) was found entangled around the base of the sigmoid colon (Figure 3). The distal ileum, caecum, and proximal ascending colon were found gangrenous. Resection of the gangrenous bowel with double barrel ileocolostomy, Hartmann procedure and distal transverse colon mucus fistula on the left side (Figure 4) was done in view of hemodynamic instability. A transverse colon mucus fistula was created to assess the bowel viability as the patient was on ionotropic support.

**Figure 5:**
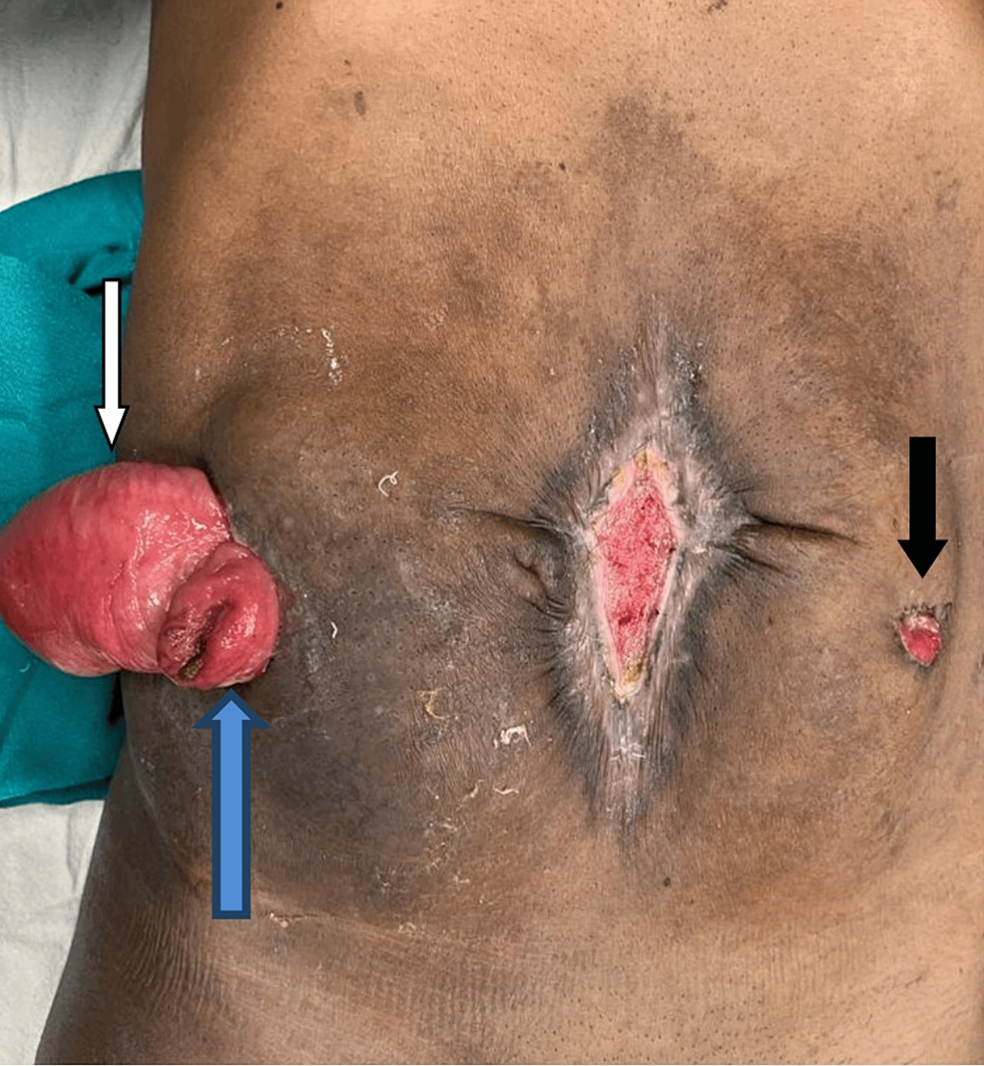
The patient at 14 weeks of surgery. End ileostomy (blue arrow), transverse colon mucus fistula (white arrow) on the right side with descending colon mucus fistula (black arrow) on the left side.

The patient was taken for stoma reversal after 16 weeks after an adequate gain in body weight. A preoperative distal loopogram (Figure 5) was done which revealed normal bowel loops without any contrast extravasation from the rectal stump. Using a midline approach ileocolic and colorectal anastomosis was done by 75 mm G.I. stapler and circular stapler respectively. The patient was discharged after eight days of hospital stay without any significant post-operative complications.

## Discussion

The ISK is a type of closed-loop intestinal obstruction that is uncommon but potentially fatal. ISK's exact mechanism is currently unknown [5,6]. Predisposing anatomical variables include a large small bowel mesentery and a narrow sigmoid pedicle. The illness mostly affects adults in their thirties and forties, with a mean age of 35.8 to 55. ISK is primarily a male condition for unknown causes, similar to primary small bowel volvulus and sigmoid volvulus. Usually, patients with ISK present significantly earlier. However, delayed presentation is not unusual. According to the literature, the average symptom duration at presentation ranged from 0.9 to 4.4 days [7-9]. Abdominal pain or tenderness, vomiting, inability to pass flatus or feces, abdominal distention, a hypoactive bowel sounds are the main symptoms of ISK upon presentation. Up to 60% of patients with ISK present with shock [8-9]. In a patient with clinical indications of bowel obstruction, the presence of features of double intestinal obstruction and dilated large and small bowel loops with multiple air-fluid levels should be a strong diagnostic of the potential diagnosis of ISK. Strangulation rates in the literature range from 73.5 to 93.9% and both segments are frequently affected (the ileum and sigmoid) [9].

Four types of ileosigmoid knotting are summarized in Table 1. In the present case, we have a type 2B knot with 360° of sigmoid over ileocecum. A new classification of ileosigmoid knotting is described in Table 2. When a patient presents with similar complaints, management should start with urgent intravenous fluid resuscitation and stabilization. Due to the high prevalence of bowel gangrene and concomitant bacterial translocation, empiric antibiotic therapy should be started as soon as possible and continued following surgery, especially in gangrenous bowel [10-11]. The presence of shock, surgical morbidity and laparotomy findings should all be taken into consideration for choice of procedure [12]. A definitive procedure may be performed during the initial laparotomy in patients who remain hemodynamically stable during the surgery. If the portion of the small bowel implicated in the knot is extensive and of doubtful viability with a risk of short gut syndrome if resected, a secondary laparotomy may be required [13].

**Table 1:** Type of ileosigmoid knotting

S. no.	Type of ISK	Percentage	Active component	Passive component	Type of rotation
1	Type 1A, Type 1B	53.9–57.5	Ileum	Sigmoid colon	Clockwise Anticlockwise
2	Type 2A, Type 2B	18.9–20.65	Sigmoid colon	Ileum	Clockwise Anticlockwise
3	Type 3	1.5	Ileocaecal segment	Sigmoid colon	—
4	Type 4	—	Undetermined	—	—

**Table 2:** New classiﬁcation for ISK C: class; A (age): A0: under 60 years; A1: 60 years and older; D (associated disease): D0: absent; D1: present; S (shock): S0: absent; S1: present; G (bowel gangrene): G0: absent; G1: present in the ileum or sigmoid colon; G2: in both segments.

C1	C2a	C2b	C3a	C3b	C4a	C4b	C5	C6
A0	One of A, D1	Two of A, D1	At most 1 of A, D1	Two of A, D1	At most 1 of A, D1	Two of A, D1	—	—
D0 S0	S0	S0	S1	S1	S0	S0	S1	—
G0	G0	G0	G0	G0	G1	G1	G1	G2

ISK is an uncommon entity with a poor prognosis. For each of these cases, a high level of suspicion is necessary. An immediate exploratory laparotomy is the best option for a better outcome in consideration of the diagnostic ambiguity and the fatal course of the disease.

## Conclusions

ISK is a less-known cause of intestinal obstruction that is frequently associated with bowel gangrene and high mortality. In order to effectively treat this condition, an emergency laparotomy along with a good pre- and post-operative care is required. Most cases of compound volvulus can be safely treated with damage control surgery.
